# Self-Healing Microcapsule-Thickened Oil Barrier Coatings

**DOI:** 10.34133/2019/3517816

**Published:** 2019-01-27

**Authors:** Alane Tarianna O. Lim, Chenlong Cui, Hee Dong Jang, Jiaxing Huang

**Affiliations:** ^1^Department of Materials Science and Engineering, Northwestern University, Evanston, Illinois 60208, USA; ^2^Rare Metals Research Center, Korea Institute of Geoscience and Mineral Resources, Daejeon 305-350, Republic of Korea

## Abstract

Low-viscosity oils could potentially act as self-healing barrier coatings because they can readily flow and reconnect to heal minor damage. For the same reason, however, they typically do not form stable coatings on metal surfaces. Increasing viscosity helps to stabilize the oil coating, but it also slows down the healing process. Here, we report a strategy for creating highly stable oil coatings on metal surfaces without sacrificing their remarkable self-healing properties. Low-viscosity oil films can be immobilized on metal surfaces using lightweight microcapsules as thickeners, which form a dynamic network to prevent the creep of the coating. When the coating is scratched, oil around the opening can rapidly flow to cover the exposed area, reconnecting the particle network. Use of these coatings as anticorrosion barriers is demonstrated. The coatings can be easily applied on metal surfaces, including those with complex geometries, both in air or under water, and remain stable even in turbulent water. They can protect metal in corrosive environments for extended periods of time and can self-heal repeatedly when scratched at the same spot. Such a strategy may offer effective mitigation of the dangerous localized corrosion aggravated by minor imperfections or damage in protective coatings, which are typically hard to prevent or detect, but can drastically degrade metal properties.

## 1. Introduction

Barrier coatings can retard corrosion by isolating underlying metal from reactive environments [1]. Pinholes and other minor damage (e.g., cracks and scratches) in the coating only expose small areas of metal. However, reactions at these sites can develop into dangerous localized corrosion, which can lead to catastrophic failure of the overall material system even with very little mass loss [2, 3]. Since these defects are hard to prevent, predict, or detect, responsive coatings that can self-repair would be very useful for mitigating localized corrosion. There have been a number of strategies for making self-healing coatings that can fix damage autonomously [4]. At the molecular level, systems with reversible bonding [5] can be triggered to reestablish connections, preventing cracks from propagating. Repairing larger cracks requires the delivery of healing agents to damaged areas. For example, microcapsules [6] and vascules [7] containing monomers and initiators can be embedded in a polymer coating, which, upon rupture, release the liquids to rapidly fill a crack, polymerize, and solidify.

Since fluid readily flows and reconnects, materials with such liquid-like properties would be ideal for self-healing purposes. However, for the same reason, low-viscosity fluid does not form stable coatings. On the other hand, high-viscosity liquid (e.g., a grease) can form very stable coatings, but it does not flow easily to heal scratched areas. Continuous layers of low-viscosity oils can be stabilized on a surface with patterned pinning sites, which essentially restructures the liquid films into interconnected small reservoirs. These oil films can then act as protective barriers to isolate the substrate from water [8–12]. Here, we report another strategy for fabricating liquid-based self-healing coatings, in which low-viscosity oil films are immobilized on metal surfaces by a dynamic network of lightweight colloidal capsules. At the macroscopic scale, the oil coating is thickened and becomes creep-resistant on metal surfaces. But, at the microscopic scale, the oil trapped within the particle network is still highly fluidic and can readily flow and reconnect when the network is broken by a scratch, thus retaining the extraordinary self-healing capability of the oil. The coatings can be applied on demand on metal surfaces as anticorrosion barriers, even under water. They are pinhole-free and stable in high turbulence and highly corrosive environments and can self-heal up to hundreds of times, making them a promising option for underwater anticorrosion applications.

## 2. Results and Discussion

### 2.1. Microcapsule-Thickened Oil

**Figure 1:**
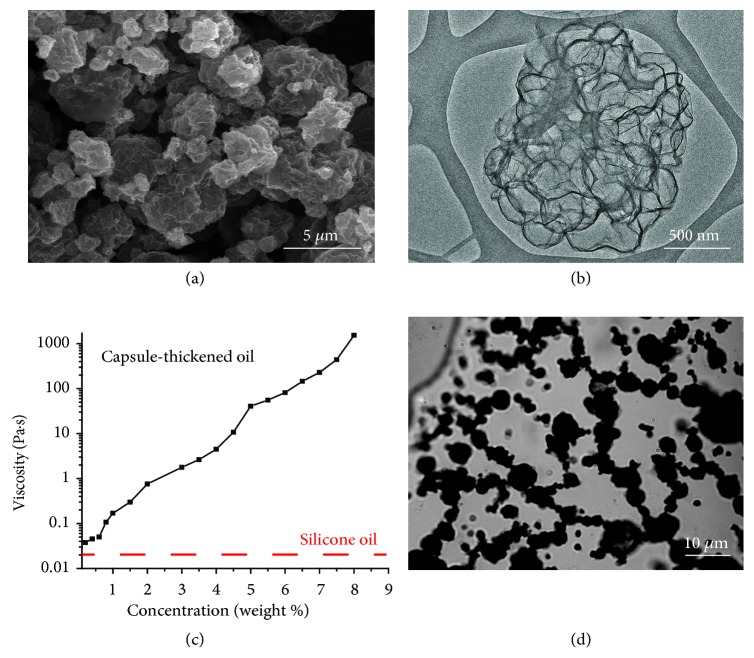
**Low-viscosity oil thickened by r-GO microcapsules. **(a) SEM and (b) TEM images of the r-GO capsules. (c) Effect of particle loading on the viscosity of a low-viscosity silicone oil (red, dashed line). (d) An optical microscopy image taken near the edge of a thickened oil film on glass slide, confirming a network structure of r-GO capsules. Thicker parts of the film are hard to image due to overlapping particles.

Oil can be gelled or thickened by molecule-, polymer-, or particle-based additives, which form an extended network in oil to stop its free flow [13]. Since barrier coatings are often applied to vertical surfaces, lightweight, low-loading-level particle thickeners would be preferred, as they do not significantly increase the weight of the oil layer. Thus, hollow microcapsules of reduced graphene oxide (r-GO) with an apparent density of around 0.12 g/cm^3^ (Figures 1(a) and 1(b)) are chosen as the model particle thickener for all the studies below. The microcapsules are made by spray-drying a mixture of graphene oxide sheets and polystyrene colloids around 200 nm in diameter, followed by thermal annealing to reduce graphene oxide and remove the polystyrene beads (see Materials and Methods). The resulting microcapsules are made of interconnected voids of around 200-250 nm in diameter with thin graphene walls of less than 10 nm. They are sufficiently robust and resilient during handling. As shown in Figure 1(c), r-GO microcapsules can increase the viscosity of silicone oil by 1000 times at just about 5 wt. % loading. Optical microscopy observation confirmed that the r-GO microcapsules indeed form an extended network in the oil (Figure 1(d)). Heavier hollow microcapsules made of poly(*o*-methoxyaniline) or silica were also tested, but much higher loading levels (e.g., 15-35 wt. %) are required for the resulting coatings to achieve similar increases in viscosity. The drastic thickening effect of r-GO microcapsules is primarily attributed to their light weight. r-GO microcapsules also have a few other desirable properties. Our earlier work [14] demonstrated that similarly prepared r-GO capsules could absorb oil well, allowing them to stay wetted by and immersed in the oil rather than floating on the surface. The black color of r-GO also facilitates direct visual inspection and optical microscopy observation of the oil coating.

### 2.2. Stability of r-GO/Oil Coating on Metal Surfaces

**Figure 2:**
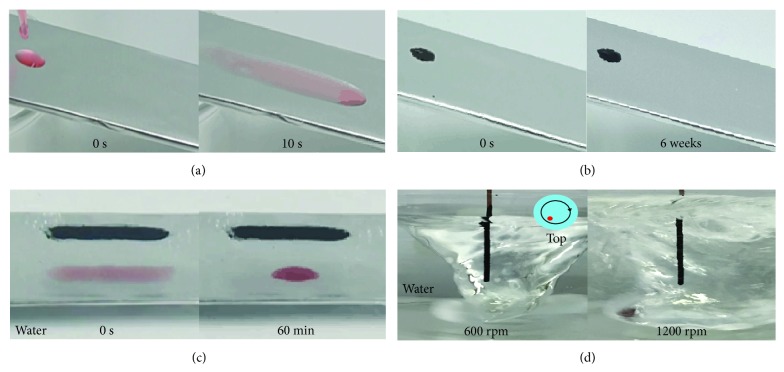
**Stability of r-GO/oil coating on metal. **(a) Photos showing a drop of dyed silicone oil (viscosity: around 0.02 Pa·s) flowing down a tilted Al foil in only 10 seconds. (b) In contrast, a drop of r-GO/oil is firmly pinned. (c) Photos showing coatings of r-GO/oil (top) and a high-molecular-weight silicone oil (bottom) of even higher viscosity (around 100 Pa·s), which is dyed red for the convenience of observation. Both coatings are initially stable in air, but when placed under water, the silicone oil film dewets and shrinks within 60 minutes. In contrast, the r-GO/oil coating remains stable. (d) The r-GO/oil coating on a copper wire (1.02 mm diameter) remains stable in a vigorously stirred water bath for days. The inset indicates the position of the wire in the bath.

Figure 2 shows that coatings made from the r-GO thickened oil are remarkably stable in air and under water. As shown in Figures 2(a) and 2(b), a drop of low-molecular-weight silicone oil with viscosity of around 0.02 Pa·s readily flows down a slope of Al foil (Figure 2(a)), while the same oil loaded with r-GO capsules (around 5 wt. %, hereafter denoted as r-GO/oil) sticks to the foil firmly (Figure 2(b)). The thickened oil has a viscosity of around 40 Pa·s. In a control experiment, high-molecular-weight silicone oil with even higher viscosity (around 100 Pa·s) was tested, which can also form a stable coating on Al foil. However, when immersed under water, the high-viscosity oil film gradually dewets (Figure 2(c)) due to the added interfacial tension between oil and water. In contrast, the r-GO/oil coating remains stable. The r-GO/oil coating is capable of resisting lateral compressive stresses induced by the surface tension of water and hinders the shrinkage of the oil film, which may be attributed to the jamming of the particles. The r-GO/oil coating can withstand highly turbulent water. Figure 2(d) shows a coated copper wire immersed in a whirlpool generated by magnetic stirring, from 600 rpm to the maximum stirring speed of 1200 rpm. The coating remains intact after days of vigorous stirring. Under these stirring conditions, the linear velocities of water around the wire are estimated to be in the range of 0.5 to 1 m/s using a dye-tracking method (see Materials and Methods), which are on par with the typical flow rates of rivers [15].

### 2.3. Barrier Performance of r-GO/Oil Coating

**Figure 3:**
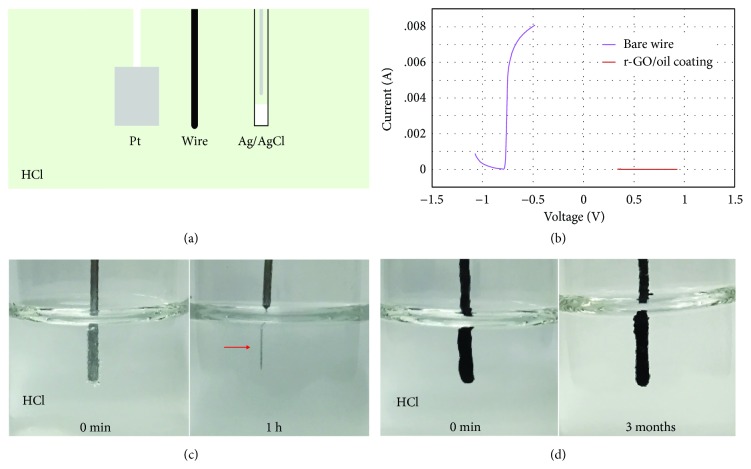
**r-GO/oil barrier coating protects metal against corrosion. **(a) Schematic drawing illustrating a 3-electrode electrochemical setup to evaluate the r-GO/oil film's anticorrosion performance in 1 M (3%) HCl. (b) Potentiodynamic polarization curves of uncoated (purple line) and coated (red line) Al wires, showing that the r-GO/oil film indeed acts as a barrier and prevents the underlying Al from reacting with HCl. (c) An Al wire (1.02 mm diameter) is severely etched after just one hour in concentrated HCl (5.5 M or 17% HCl). (d) In contrast, an Al wire coated with r-GO/oil film remains intact after at least 3 months.

The r-GO/oil coating can indeed act as a barrier to protect metal against corrosion over extended periods of time. A 3-electrode electrochemical cell (Figure 3(a)) consisting of platinum as the counter electrode, an Al wire as the working electrode, and Ag/AgCl as the reference electrode was used to evaluate the barrier performance of the r-GO/oil film in a solution of 1 M (3%) HCl. The potentiodynamic polarization curve of a bare Al wire (Figure 3(b), purple line) shows anodic and cathodic branches typically associated with the corrosion of a metal in a solution. In contrast, the same experiment performed on an r-GO/oil-coated Al wire (Figure 3(b), red line) results in a nearly flat line around zero current, indicating that the r-GO/oil coating insulates the underlying Al from reacting with the electrolyte solution and thus prevents the metal from corroding. Figures 3(c) and 3(d) show the long-term anticorrosion performance of the r-GO/oil coating. An Al wire immediately starts to react upon dipping in 20% HCl solution (Figure 3(c), left), generating H_2_ bubbles on its surface. After one hour, the immersed part of the wire is almost entirely etched (Figure 3(c), right). However, an r-GO/oil-coated wire (Figure 3(d), left) stays intact after being immersed in this highly corrosive solution for at least 3 months (Figure 3(d), right). Some coated wires were found to be intact after being immersed for over a year.

**Figure 4:**
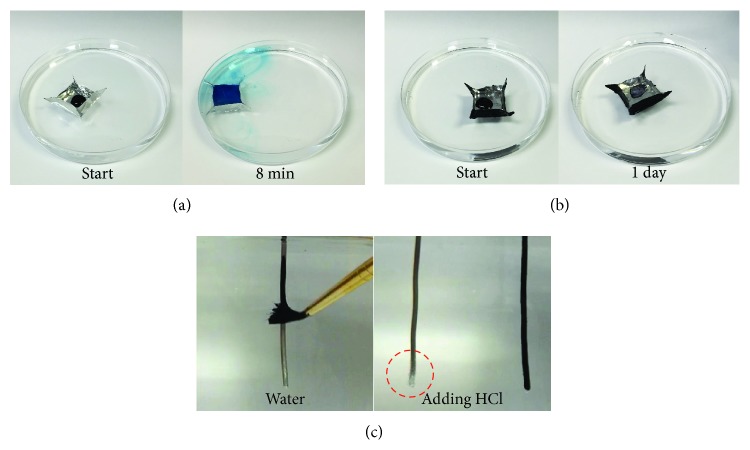
**r-GO/oil can form stable coating on metal of complex geometry and can be applied from underwater. **(a) An Al foil boat placed on 2 M HCl solution is damaged after just 8 minutes, indicated by the leakage of methylene blue solution, and completely dissolves away after 20 minutes. (b) Another Al boat coated with an r-GO/oil film stays intact after a day, when the dye solution has dried out, showing that the coating is stable on metal surface with complex geometry and sharp corners. (c) r-GO/oil film can be brushed onto an already immersed Al wire under water to protect it from reacting with concentrated HCl. As a control, a bare Al wire is also immersed, which immediately starts to bubble (dashed red circle) due to reaction with HCl. Also see [Supplementary-material supplementary-material-1].

The r-GO/oil film adheres well to many types of metal surfaces (e.g., Cu, Fe, Al, and their alloys), even those with complex geometries or sharp corners, on which oil film tends to dewet. An example is demonstrated in Figures 4(a) and 4(b), showing the survival tests of an Al foil boat placed on a sea of 2 M HCl. The boats are loaded with a methylene blue dye solution to indicate leakage. Without a barrier coating, the Al boat is rapidly etched by HCl. It starts to leak after a few minutes and completely dissolves in 20 minutes (Figure 4(a)). In contrast, the boat coated with an r-GO/oil film is well protected and remains intact after the dye solution (Figure 4(b)) or even the entire HCl bath dries out. The r-GO/oil coating can be conveniently applied to metal surfaces on demand, even under water, simply with a brush to yield a pinhole-free barrier coating (see photos in Figure 4(c) and [Supplementary-material supplementary-material-1]) that is capable of stopping ongoing corrosion.

### 2.4. Self-Healing Property of r-GO/Oil Coating

**Figure 5:**
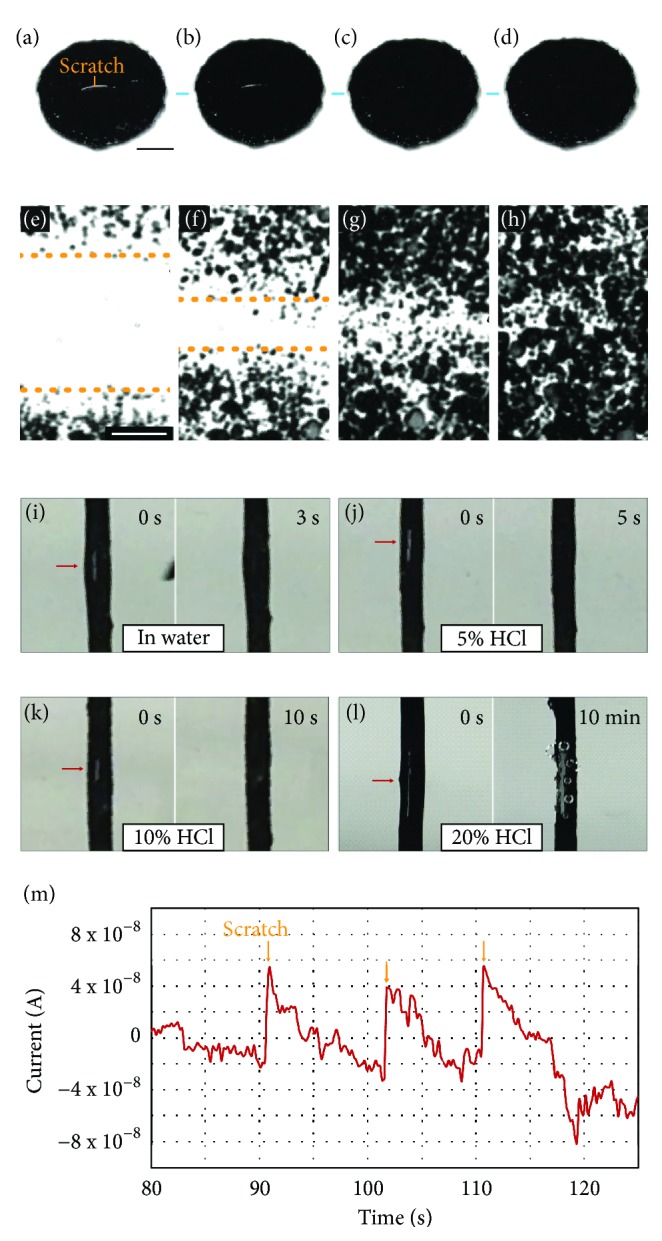
** Self-healing property of r-GO/oil coating. **((a)-(d)) Snapshots showing that a scratch about 0.5 mm wide fully heals in seconds (scale bar = 0.5 mm). ((e)-(h)) The corresponding optical microscopy images reveal that oil can rapidly flow to the scratched area, followed by reorganization of particles to reestablish the network (scale bar = 50 *μ*m). ((i)-(l)) Self-healing of r-GO/oil coating when scratched in (i) water, (j) 5% HCl, (k) 10% HCl, and (l) 20% HCl. The position of the scratch is indicated by the red arrows. The healing time in more concentrated HCl solution increases since the flow of oil to the exposed area is increasingly hindered by H_2_ evolution at faster etching rate. In 20% HCl, the rate of H_2_ evolution is too fast to allow self-healing to proceed. (m) Open-circuit current of an Al wire coated with r-GO/oil immersed in 1 M (3%) HCl during a scratch test. When the coating is scratched, a small area of the metal is exposed. The resulting local corrosion triggers a spike in the current that quickly dissipates within a few seconds, indicating that the coating has self-healed to restore its protective barrier property.

While the r-GO/oil coating exhibits remarkable stability, it does not lose the self-healing properties of the oil. The coating is capable of healing submillimeter to millimeter scale scratches in seconds, as shown in the snapshots in Figures 5(a)–5(d) (also see [Supplementary-material supplementary-material-1]). Optical microscopy observation (Figures 5(e)–5(g), also see [Supplementary-material supplementary-material-1]) reveals that when a scratch breaks part of the particle network nearby, oil immediately starts flowing to the exposed area and brings new particles to reestablish the network. The flow of particles stops after the coating is healed (Figure 5(h)). Figures 5(i)–5(l) show coatings that have been applied to aluminum wires. They demonstrate that the coating can quickly self-heal in both water (Figure 5(i)) and in 5% and 10% HCl (Figures 5(j) and 5(k), respectively) when scratched. Eventually, when immersed in 20% HCl solution, the evolution of H_2_ bubbles at the scratched area is too fast to allow the r-GO/oil coating to recover (Figure 5(l)). This self-healing behavior can also be seen in open-circuit current measurements (Figure 5(m)) of an Al wire that is coated with r-GO/oil and immersed in 1 M (3%) HCl. When the coating is scratched, a small area of the metal is exposed, triggering a current spike that gradually decays to near zero within a few seconds, indicating that the coating has self-healed. The coating can self-heal multiple times in succession. The duration of the current spikes matches the time scale of the self-healing behaviors observed in Figures 5(a)–5(d).

**Figure 6:**
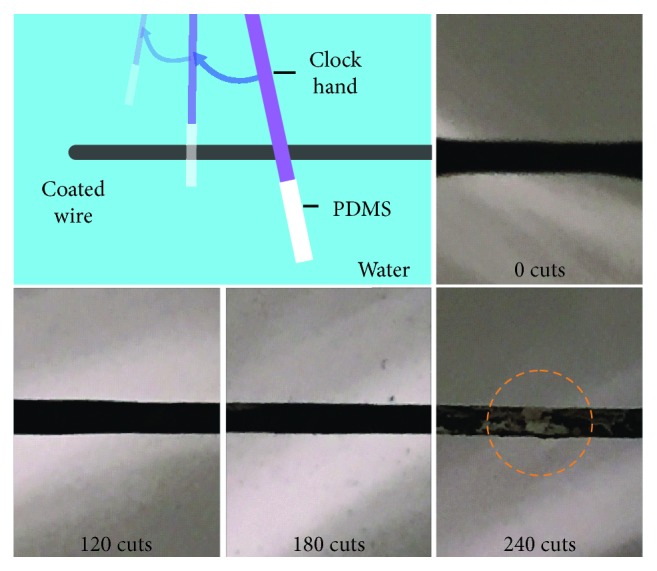
**Exhaustive scratching and healing test.** As shown in [Supplementary-material supplementary-material-1], a PDMS rod with a diameter of around 1 mm is attached to a clock hand to repeatedly scratch an r-GO/oil-coated wire, at the same spot, once every minute. Every scratch removes a small piece of the coating, which repeatedly heals until there is insufficient amount of r-GO/oil left to form a complete coverage on the wire. For the wire (1.02 mm in diameter) shown in the photo, the coating sustained 180 scratches. Unhealable damage on the coating becomes visible after 240 scratches.

Although only 3 consecutive scratching-healing cycles were shown in the electrochemical test shown in Figure 5(m), the r-GO/oil coating is actually quite tolerant to scratches and can self-heal up to hundreds of times at the same spot. The drawing in Figure 6 illustrates an exhaustive scratch test on an r-GO/oil-coated wire immersed under water. A soft rod made of polydimethylsiloxane (PDMS) is attached to the minute hand of a clock, so that it can repeatedly scratch the immersed wire at the same location once every minute. As long as there is a sufficient reserve of oil to flow to the scratched area and extra capsules above the percolation threshold to reconnect the broken network, the r-GO/oil coating sustains scratching and self-heals repeatedly (also see [Supplementary-material supplementary-material-1]). Therefore, although a small piece of the coating is removed during each scratch, the coating shown in Figure 6 repeatedly heals even after 180 scratches. After 240 scratches, the damage on the coating becomes visible, when the remaining amount of r-GO/oil becomes insufficient to completely cover the wire.

### 2.5. Mitigation of Localized Corrosion with Self-Healing Coating

**Figure 7:**
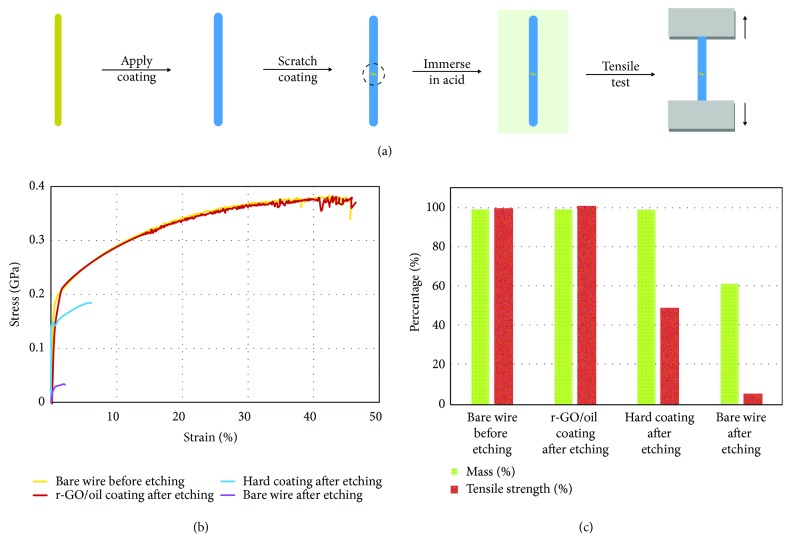
**The r-GO/oil coating is scratch-tolerant and protects metal wires from localized corrosion. **(a) Schematic illustration of the experimental procedure testing the effects of localized corrosion on the mechanical properties of brass wires. A wire is first coated with a barrier film and then scratched to expose a small area before being immersed in etching solution (5.5 M or 17% HCl). After etching, tensile tests are performed to directly evaluate corrosion-induced damage. (b) Representative stress-strain curves of an unetched wire, etched wire, etched wire with an unhealable coating, and etched wire with the r-GO/oil coating. (c) A bar graph summarizing changes in tensile strength and the mass of the wires tested in (b). The unprotected wire loses nearly 40% of mass and over 90% of strength after 1 week. The polymer-coated wire has negligible mass loss, even after 2 weeks, but its strength is decreased by about half, due to localized corrosion at the scratch. The wire coated with the self-healing r-GO/oil film retains its original mechanical properties and is not affected by the scratch.

The scratch-tolerant, self-healing properties of r-GO/oil coatings make them an effective type of barrier for mitigating localized corrosion. A proof-of-concept experiment is shown in Figure 7(a), in which brass wires are first protected with a barrier coating and then scratched to expose a small area to simulate localized corrosion, before immersing them in highly corrosive solutions (5.5 M HCl) for 2 weeks. A hard polymer coating is tested as a control to illustrate the importance of the self-healing capability of the barrier coating. Typical corrosion tests often measure the mass loss of metals. Such measurements do not reflect the drastic degradation of mechanical properties by localized corrosion, which can occur with negligible mass loss [3, 16]. Therefore, tensile tests are performed to directly evaluate the damage in mechanical properties as a result of localized corrosion on the wires. Figure 7(b) shows representative stress-strain curves of a number of brass wire samples before and after etching. Figure 7(c) compares the percentages of tensile strength and mass of these wires after etching, relative to those of the unetched wire. Without a protective barrier, the wire loses nearly 40% of its mass and over 90% of its strength after just 1 week. The wire coated with a hard polymer barrier experiences negligible mass loss after 2 weeks, but its strength decreases by about 50% due to localized corrosion at the scratched area (see SEM images in [Supplementary-material supplementary-material-1]). In fact, even without the intentionally made scratch, the polymer-coated wires still suffer significant property degradation from corrosion due to pinholes, which are hard to prevent and detect (see [Supplementary-material supplementary-material-1]). In contrast, with the r-GO/oil's rapid self-healing capability, the wire coated with r-GO/oil retains its original mechanical properties even after being immersed in the etchant solution for 2 weeks.

## 3. Conclusion

In conclusion, by using lightweight microcapsules as thickening agents, even low-viscosity oil can form continuous, highly stable, protective barrier coatings on metal surfaces. Such oil coatings are intrinsically pinhole-free, and they can quickly self-heal many times when they are scratched, making them potentially useful as an on demand or urgent solution for protective barrier applications. Although most of the work presented here is demonstrated with r-GO microcapsules, the described strategy is largely materials agnostic and should be applicable to a wide range of lightweight particles. The particles can potentially be loaded with other materials to render additional functions to enhance the barrier coatings.

## 4. Materials and Methods

### 4.1. Experimental Design

#### 4.1.1. Materials

Graphene oxide (GO) sheets were synthesized through a modified Hummer's method [17] as reported elsewhere [18]. Polystyrene colloids were prepared through emulsion polymerization [19]. Reduced graphene oxide (r-GO) capsules are made by an aerosol-assisted synthesis method based on a previous report [14], using a spray dryer (Buchi Nano-Spray Dryer B-90). A mixture of 1 L 2 mg/mL GO sheets and 100 mL polystyrene colloids (200 nm diameter) was sprayed at 80 °C, which yielded GO-wrapped polystyrene beads. r-GO capsules were obtained by heating the product under argon at 600 °C for 4 hours, which reduced GO and removed the sacrificial polymer template. The apparent density of the capsules was determined to be 0.12 g/cm^3^ by measuring the volume of a known mass of powder within the end of a cylindrical pipette tip. SEM images of the r-GO capsules were taken with an FEI Nova 600 SEM. TEM images were taken with a JEOL ARM300F GrandARM transmission electron microscope. These particles were added to oil at various weight fractions to adjust viscosity. Various types of oils such as household vegetable oils, household sunscreen oils, light mineral oils, and silicone oils were tested, all of which worked for self-healing coatings. Silicone oil was chosen as the model system due to its high stability against degradation and low solubility in water. Low-molecular-weight (viscosity 20 cSt, i.e., around 0.02 Pa·s) and high-molecular-weight (viscosity 100,000 cSt, i.e., around 100 Pa·s) silicone oils were purchased from Sigma-Aldrich. A number of metal wires were tested, including brass, copper, steel, and aluminum. The wires were briefly polished with sandpaper and washed with ethanol to remove any existing surface coating. Hollow spheres of poly(*o*-methoxy)aniline (average diameter: 2.27 *μ*m, average wall thickness: 191 nm) and silica (average diameter: 3.94 *μ*m, average wall thickness: 223 nm) were synthesized using methods in the literature [20, 21].

#### 4.1.2. Viscosity Measurement (Figure 1(c))

Viscosities of the r-GO/oil coatings were measured on an Anton Paar Physica MCR 300 rheometer with a cone-and-plate (lower loading levels) or parallel plate geometry (higher loading levels). Typically, 0.5 g of particle/oil coating was subjected to shear rates from 0.1 to 100 rad/s to measure the resulting shear stresses. The viscosity at 0.1 rad/s was chosen for comparison.

#### 4.1.3. Stability under Shearing Water (Figure 2(d))

Copper wires (1.02 mm diameter) coated with r-GO/oil films were immersed in a water bath, which was stirred at a nominal speed of 600 and 1200 rpm for 1-2 weeks. The linear shear velocity of water is estimated to be 0.5 to 1 m/s using a dye-tracking method, in which a droplet of concentrated dye solution is dispensed into the whirlpool and tracked using a camera in slow motion mode (240 fps). The initial linear velocity of this droplet (e.g., within the first 100 ms, before it becomes too diffuse) was calculated to represent the linear flow rate of the whirlpool.

#### 4.1.4. Electrochemical Tests (Figures 3(a), 3(b), and 5(m))

The anticorrosion performance of r-GO/oil on aluminum in a 1 M (3%) HCl solution was evaluated using an Autolab electrochemical interface instrument (PGSTAT 302N). The electrochemical cell (illustrated in Figure 3(a)) was a three-electrode setup consisting of platinum (counter electrode), a freshly polished aluminum wire that was either bare or coated with r-GO/oil (working electrode) and Ag/AgCl (reference electrode). The polarization curves (Figure 3(b)) were measured from -0.3 V_OCP_ to 0.3 V_OCP_ at a scan rate of 0.001 V/s and a step size of 0.01 V. To investigate the electrochemical behavior of self-healed scratches (Figure 5(m)), open-circuit current of an r-GO/oil-coated Al wire immersed in the same electrochemical cell was monitored at a time increment of 0.2 s. The wire was scratched with a plastic pipette tip to induce local corrosion.

#### 4.1.5. Anticorrosion Tests (Figures 3(c), 3(d), 4(a), and 4(b))

An uncoated Al wire (1.02 mm diameter) (Figure 3(c)) and another coated with r-GO/oil film (Figure 3(d)) were immersed into 5.5 M (17%) HCl. Al boats shown in Figures 4(a) and 4(b) were made from foil by folding and floated on a solution of 2 M HCl in a 100 mm diameter petri dish. 0.2 mL of 0.1 wt. % methylene blue solution was loaded in the boats as color indicator of leakage.

#### 4.1.6. Visual and Optical Microscopy Observation of Self-Healing Property (Figure 5, Movies [Supplementary-material supplementary-material-1] and [Supplementary-material supplementary-material-1])

r-GO/oil coating was applied onto a glass slide and swiped with a 200 *μ*L pipette tip to generate scratches that are about 0.5 to 1 mm wide. Optical microscopy images (Nikon Eclipse TE2000-U) were recorded using a monochrome interline CCD camera (Photometrics, CoolSNAP HQ2).

#### 4.1.7. Exhaustive Self-Healing Test (Figure 6 and [Supplementary-material supplementary-material-1])

A metal wire coated with r-GO/oil was fastened horizontally under water. A polydimethylsiloxane (PDMS) rod with diameter around 1 mm was used to scratch the coating repeatedly at the same spot. The PDMS rod was attached to the “second” hand of a ticking clock, so that it scratched the coated wire once per minute.

#### 4.1.8. Evaluating Corrosion-Induced Degradation of Mechanical Properties (Figures 7, [Supplementary-material supplementary-material-1] and [Supplementary-material supplementary-material-1])

Brass wires were first coated with r-GO/oil or Rust-Oleum 2X (a polymer-based anticorrosion paint) and then scratched with a razor blade to generate small slits that are around 0.3 mm wide. Wires with scratched coatings were immersed into 5.5 M (17%) HCl (1 week for uncoated wires, 2 weeks for coated wires). Stress-strain curves were obtained using a Bose ElectroForce 5500 tensile tester. SEM images of the wire surfaces after corrosion were taken with an FEI Nova 600 SEM. In control experiments, wires coated with the paint, but unscratched, were also immersed in HCl to show the effect of pinholes, which are hard to prevent and detect during the coating process.

## Supplementary Materials

Supplementary Materials Fig.  S1.** SEM images of etched brass wire samples used in Fig. 7.**  (a) The surface of a new wire is relatively smooth before etching. (b) After etching in 5.5 M (17%) HCl for 7 days, the surface is highly corroded and roughened. (c) A wire protected by the r-GO/oil coating shows no signs of corrosion even after 14 days of etching. (d) Significant, localized corrosion occurs at the scratched area of the brass wire protected by the polymer coating.** Fig. S2. Localized corrosion through pinholes can drastically degrade the properties of metal wires.**  (a) Stress-strain curve of a brass wire protected with an unscratched polymer coating after being immersed in 5.5 M (17%) HCl for 14 days, compared to that of a pristine wire. The etched wire experiences no detectable mass loss, but obvious reduction in strength and ductility. (b) SEM examination of the etched wire reveals microcracks on the wire, likely due to localized corrosion through some hard-to-detect pinholes on the coating.** Movie S1**. Brushability of r-GO/oil coating under water. An r-GO/oil film is readily applied to an aluminum wire under water with a paintbrush. After the wire is completely coated, another aluminum wire is fastened next to the coated wire and HCl is added. The uncoated wire reacts with the added HCl, as shown by the formation of bubbles on the wire surface, while the coated wire remains protected.** Movie S2**. Macroscopic visualization of self-healing r-GO/oil coating. An r-GO/oil film on a glass microscope slide is scratched with a pipette tip and observed to heal within seconds. This healing process can be readily seen by eye.** Movie S3**. Microscopic visualization of self-healing r-GO/oil coating. A scratch breaks the particle network within the coating. As the freed low-viscosity oil flows into the scratch, it carries lightweight microcapsules towards the scratch, which reorganize to reestablish the network.** Movie S4**. Repeatability of self-healing. A strip of PDMS attached to the “second” hand on a clock scratches a self-healing coating at one spot. The clock can be left running to test the number of times the coating can be scratched at the same location.

## References

[1] Austin M. J. (1993). *Surface Coatings*.

[2] Baboian R. (2005). *Corrosion Tests and Standards: Application and Interpretation*.

[3] Cui C., Lim A. T. O., Huang J. (2017). A cautionary note on graphene anti-corrosion coatings. *Nature Nanotechnology*.

[4] Blaiszik B. J., Kramer S. L. B., Olugebefola S. C., Moore J. S., Sottos N. R., White S. R. (2010). Self-healing polymers and composites. *Annual Review of Materials Research*.

[5] Chen X., Dam M. A., Ono K. (2002). A thermally re-mendable cross-linked polymeric material. *Science*.

[6] White S. R., Sottos N. R., Geubelle P. H. (2001). Autonomic healing of polymer composites. *Nature*.

[7] Toohey K. S., Sottos N. R., Lewis J. A., Moore J. S., White S. R. (2007). Self-healing materials with microvascular networks. *Nature Materials*.

[8] de Gennes P.-G., Brochard-Wyart F., Quéré D. (2004). *Capillarity and Wetting Phenomena: Drops, Bubbles, Pearls, Waves*.

[9] Liu M., Wang S., Jiang L. (2017). Nature-inspired superwettability systems. *Nature Reviews Materials*.

[10] Liu M., Wang S., Wei Z., Song Y., Jiang L. (2009). Bioinspired design of a superoleophobic and low adhesive water/solid interface. *Advanced Materials*.

[11] Wong T.-S., Kang S. H., Tang S. K. Y. (2011). Bioinspired self-repairing slippery surfaces with pressure-stable omniphobicity. *Nature*.

[12] Kim P., Kreder M. J., Alvarenga J., Aizenberg J. (2013). Hierarchical or not? Effect of the length scale and hierarchy of the surface roughness on omniphobicity of lubricant-infused substrates. *Nano Letters*.

[13] Tadros T. F. Rheology modifiers, thickeners, and gels. *Rheology of Dispersions (Wiley-VCH Verlag GmbH & Co. KGaA, 2010)*.

[14] Sohn K., Joo Na Y., Chang H., Roh K.-M., Dong Jang H., Huang J. (2012). Oil absorbing graphene capsules by capillary molding. *Chemical Communications*.

[15] Warren N. (2006). *Metal Corrosion in Boats: The Prevention of Metal Corrosion in Hulls, Engines, Rigging and Fittings*.

[16] Jones R. H. (1992). *Stress-Corrosion Cracking*.

[17] Hummers W. S., Offeman R. E. (1958). Preparation of graphitic oxide. *Journal of the American Chemical Society*.

[18] Kim F., Luo J., Cruz-Silva R., Cote L. J., Sohn K., Huang J. (2010). Self-propagating domino-like reactions in oxidized graphite. *Advanced Functional Materials*.

[19] Zou D., Derlich V., Gandhi K. (1990). Model filled polymers. I. Synthesis of crosslinked monodisperse polystyrene beads. *Journal of Polymer Science Part A: Polymer Chemistry*.

[20] Zhang L., Peng H., Sui J., Soeller C., Kilmartin P. A., Travas-Sejdic J. (2009). Self-assembly of poly(o-methoxyaniline) hollow microspheres. *The Journal of Physical Chemistry C*.

[21] Bruinsma P. J., Kim A. Y., Liu J., Baskaran S. (1997). Mesoporous silica synthesized by solvent evaporation: spun fibers and spray-dried hollow spheres. *Chemistry of Materials*.

